# Vibrational analysis on the revised potential energy curve of the low-barrier hydrogen bond in photoactive yellow protein

**DOI:** 10.1016/j.csbj.2015.10.003

**Published:** 2015-10-31

**Authors:** Yusuke Kanematsu, Hironari Kamikubo, Mikio Kataoka, Masanori Tachikawa

**Affiliations:** a Graduate School of Information Science, Hiroshima City University, 3-4-1 Ozuka-Higashi, Asa-Minami-Ku, Hiroshima 731-3194, Japan; b Quantum Chemistry Division, Yokohama City University, Seto 22-2, Kanazawa-ku, Yokohama 236-0027, Japan; c Nara Institute of Science and Technology, 8916-5 Takayama, Ikoma, Nara 630-0192, Japan

**Keywords:** Low-barrier hydrogen bond, Photoactive yellow protein, Vibrational analysis, ONIOM, PCM

## Abstract

Photoactive yellow protein (PYP) has a characteristic hydrogen bond (H bond) between *p*-coumaric acid chromophore and Glu46, whose OH bond length has been observed to be 1.21 Å by the neutron diffraction technique [Proc. Natl. Acad. Sci. 106, 440–4]. Although it has been expected that such a drastic elongation of the OH bond could be caused by the quantum effect of the hydrogen nucleus, previous theoretical computations including the nuclear quantum effect have so far underestimated the bond length by more than 0.07 Å. To elucidate the origin of the difference, we performed a vibrational analysis of the H bond on potential energy curve with O…O distance of 2.47 Å on the equilibrium structure, and that with O…O distance of 2.56 Å on the experimental crystal structure. While the vibrationally averaged OH bond length for equilibrium structure was underestimated, the corresponding value for crystal structure was in reasonable agreement with the corresponding experimental values. The elongation of the O…O distance by the quantum mechanical or thermal fluctuation would be indispensable for the formation of a low-barrier hydrogen bond in PYP.

## 1 Introduction

**Fig. 1:**
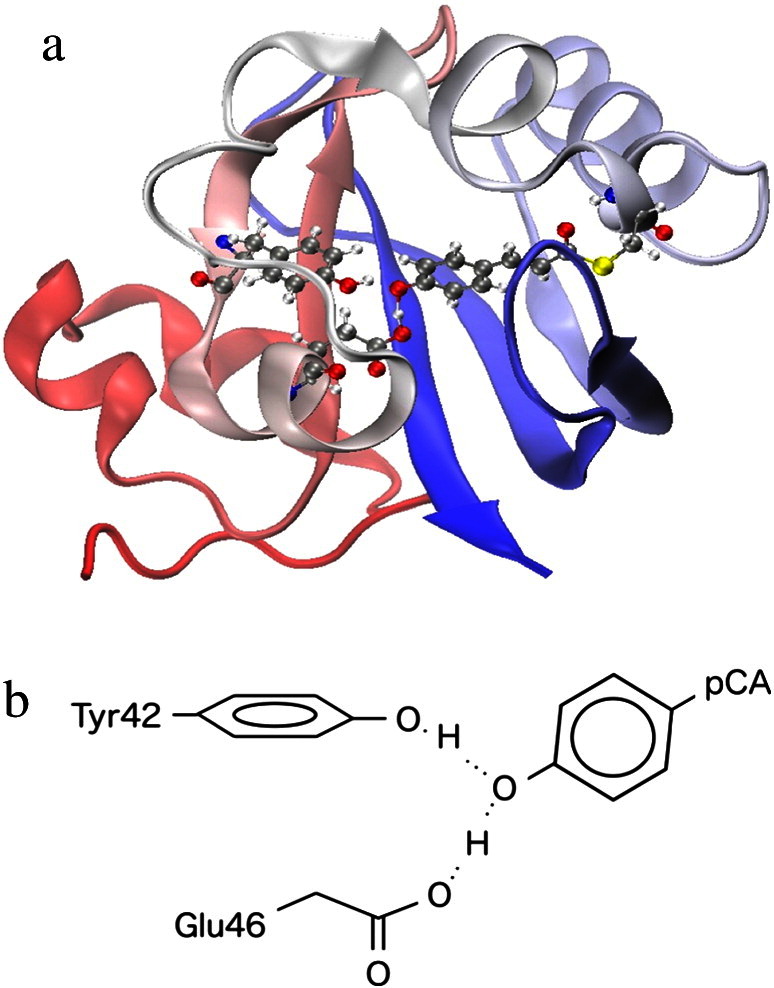
(a) The entire structure and (b) the active center of photoactive yellow protein. OH bond focused on in the present work is also indicated.

Photoactive yellow protein (PYP) is a water-soluble photosensor protein found in halophilic photosynthetic bacteria. This protein is known to play an important role in the photocycle that regulates the negative phototaxis behavior of the bacteria [1]. PYP from *H. halophila* has especially drawn substantial attention due to the characteristic hydrogen bond (H bond) in the site. Yamaguchi et al. reported the detailed crystal structure of dark-state PYP with the coordinates of hydrogen atoms by the neutron diffraction technique [2]. The structure of PYP is shown in Fig. 1. They have assigned a hydrogen bond between Glu46 and *p*-coumaric acid (pCA) chromophore of PYP with significantly long O-H bond of 1.21 Å as low-barrier hydrogen bond (LBHB), which had never been directly observed in proteins until then. They have suggested the roles of LBHB in PYP to stabilize the negative charge around the chromophore in the protein interior, and to mediate the fast proton transfer during the photocycle. It has also been found that Arg52 located near the chromophore was deprotonated (neutral), whereas it had been believed to be protonated to act as counterion for the negative chromophore according to X-ray crystallography[3] and electronic structure calculation [4].

It was, however, claimed by Saito and Ishikita that Arg52 should be protonated and the H-bond between pCA and Glu46 was not LBHB but a normal H-bond according to the potential energy profile analysis with the conventional QM/MM calculation, and the comparison between the experimental chemical shifts in solution [5] and their computational values under the previously mentioned condition [6], [7]. Hirano and Sato compared the potential energy profiles for systems with and without the protonation of Arg52 using the ONIOM method [8], [9], which is an efficient method to calculate large systems such as proteins by dividing the system into several layers, and found the low barrier height for deprotonated Arg52 model with respect to hydrogen transfer coordinates in H-bond [10].

For the computation of the molecular geometry for H-bonded systems, we should pay careful attention to the protonic or deuteronic fluctuation in H-bonds due to nuclear quantum effect [11] and not only to the electrostatic contribution of the surrounding environment. Kita et al. calculated the isolated cluster model of H-bonding center in PYP by multicomponent quantum mechanics (MC_QM) method [12], [13], [14] and found that O-H bond length becomes longer due to the nuclear quantum effect [15]. Nadal-Ferret et al. showed that long O-H bonds could be experimentally observed by taking account of the nuclear quantum fluctuation of the hydrogen nuclei, and by local vibration analysis using QM/MM for PYP with deprotonated Arg52 [16]. By comparison of the experimental and the computational geometrical parameters with and without the protonation of Arg52, they suggested that the formation of LBHB between pCA and Glu46 would be possible if Arg52 was deprotonated. Recently, we have also analyzed this geometry by the combination of ONIOM and MC_QM [ONIOM (MC_QM:MM)], and validated the above suggestion by comparison with the corresponding experimental values [17].

Table 1 lists the experimental and the computational OH bond lengths in various previous papers. Each computational work in Table 1 shows that the nuclear quantum effect of hydrogen nuclei elongates the OH bond length from the equilibrium structure. There, however, remains a difference between experimental and theoretical OH bond lengths of more than 0.07 Å, and the experimental observation was not yet fully supported by the computations.

In our previous work, we have examined several computational conditions for ONIOM calculations [17]. We found the expansion of the QM region can involve the degradation of the barrier height, which reasonably agreed with all electron ONIOM (QM:QM) calculations. It could be expected that the vibrational analysis on this potential energy curve would provide longer OH bonds than that of previous works. Therefore, the present paper will be devoted to the vibrational analysis for the model system of PYP with expanded QM region in order to elucidate the origin of the difference between the experimental measurement and the theoretical computation.

## 2 Computational detail

We performed a numerical one-dimensional vibrational analysis by solving the nuclear Schrödinger equation on the basis of the Born–Oppenheimer approximation. The analyses dealt with two initial structures of PYP, one of which was the crystal structure (PDB ID: 2ZOI; temperature, 295 K; resolution, 1.50 Å) obtained by the neutron diffraction technique [2], and the latter was the equilibrium structure that has been optimized by conventional ONIOM calculation in our previous work [17]. The missing atoms in the crystal structure have been compensated for by AmberTools [18], [19], resulting 1929 atoms with a total charge of 6– for the entire PYP. We utilized the ONIOM Electronic Embedding (ONIOM-EE) method [8], [9] with the computational condition of “System 3-dp” of ref. [17], which includes Ile31, Tyr42, Glu46, Thr50, Cys69, pCA, and Arg52 in deprotonated form inside the QM region with CAM-B3LYP/6-31 + G(d,p) [20], [21], [22] level of calculation, and the other residues are in molecular mechanical level of calculation with AMBER ff99 and GAFF parameters [23]. As in ref. [16] and the Appendix in ref. [17], unrelaxed one-dimensional potential energy curves for the migration of the hydrogen nucleus along the direction vector *q* from Glu46 to pCA have been constructed for the vibrational analysis.

## 3 Results and discussion

### 3.1 The vibrational analysis on the equilibrium structure

**Fig. 2:**
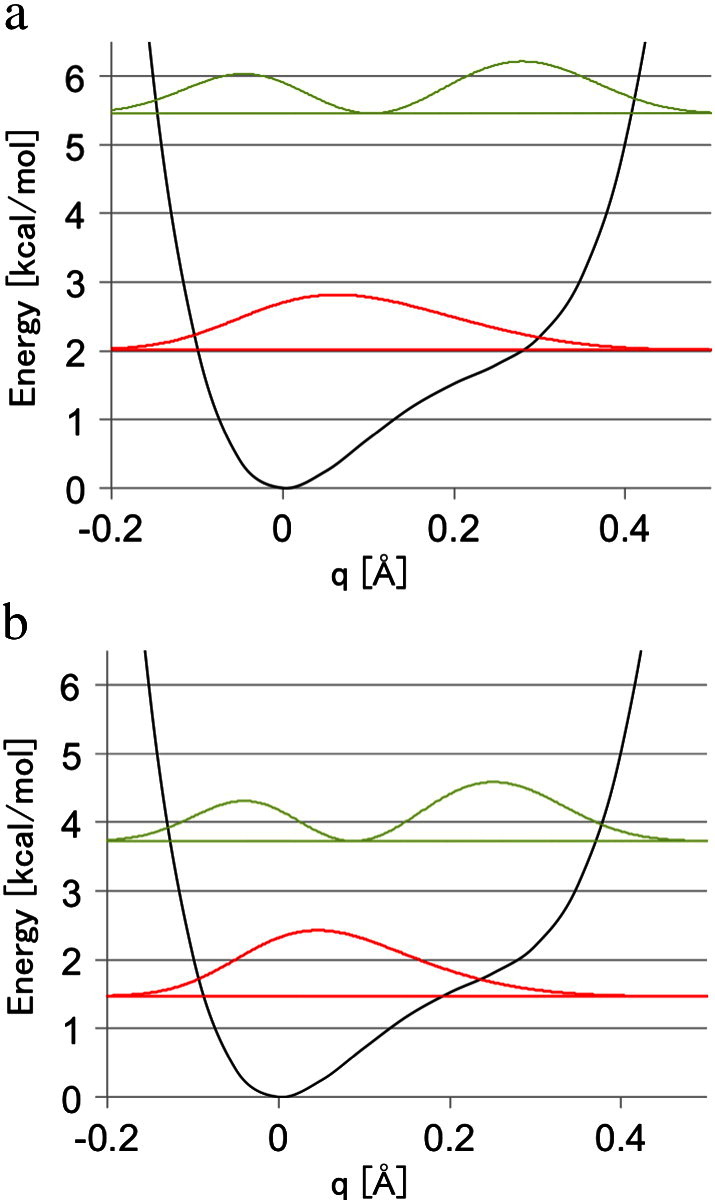
The potential energy curve (black) and the corresponding vibrational distribution of the ground (red) and the first excited (green) states of (a) a proton and (b) a deuteron in the hydrogen bond between Glu46 and pCA of PYP at the equilibrium structure. The origin of the coordinate *q* was set on the equilibrium bond length (1.08 Å).

At first, we would like to focus on the results of the vibration analysis on the equilibrium structure. Fig. 2 shows the potential energy curve and the corresponding vibrational distributions of the ground and the first excited states of a proton and a deuteron. The corresponding energy levels and the vibrationally averaged OH bond lengths are shown in Table 2. We can see the anharmonic single-well potential energy curve with no barrier, and the corresponding widespread vibrational distribution in Fig. 2. The shape of the potential energy curve was drastically different with those of the double-well curve in the previous reports [10], [16], [17]. Such difference could be mainly attributed to the short O…O distance of 2.47 Å between the H-bond donor and the acceptor, which was about 0.1 Å shorter than that of the experimental value. Table 2 indicates that the bond lengths were extended from the equilibrium lengths, as in the previous reports [10], [16], [17]. The averaged bond lengths of ground states reasonably agreed with those of our previous work with the corresponding computational condition; 1.14 Å for DD and 1.15 Å for HH isotopologues [17].

In order to include the thermal effect on the OH bond, we can utilize the Boltzmann averaging for the temperature *T* according to the equation:

(1) $$R_{T}=\frac{\sum_{i}{\langle R\rangle }_{i}\exp\left[-\epsilon_{i}/k_{B}T\right]\;}{\sum_{i}\exp\left[-\epsilon_{i}/k_{B}T\right]}\;\text{,}$$

where *ϵ*_*i*_'s are the energies of the respective vibrational states and *k*_B_ is the Boltzmann constant. We calculated the thermally averaged OH bond lengths for the first three vibrational states at room temperature (*T* = 300 K). The resulting values are 1.15 Å for a deuteron and 1.17 Å for a proton, and corresponded with those of ground states in the shown digits. This is because the excitation energies are higher than 2.0 kcal/mol, and the contributions of the excited states against the thermal averaging are less than 3% of the ground states at the room temperature. As a result, there still remained a difference of 0.06 Å from the experimental value even though the thermal effect was included.

### 3.2 The vibrational analysis on the crystal structure

**Fig. 3:**
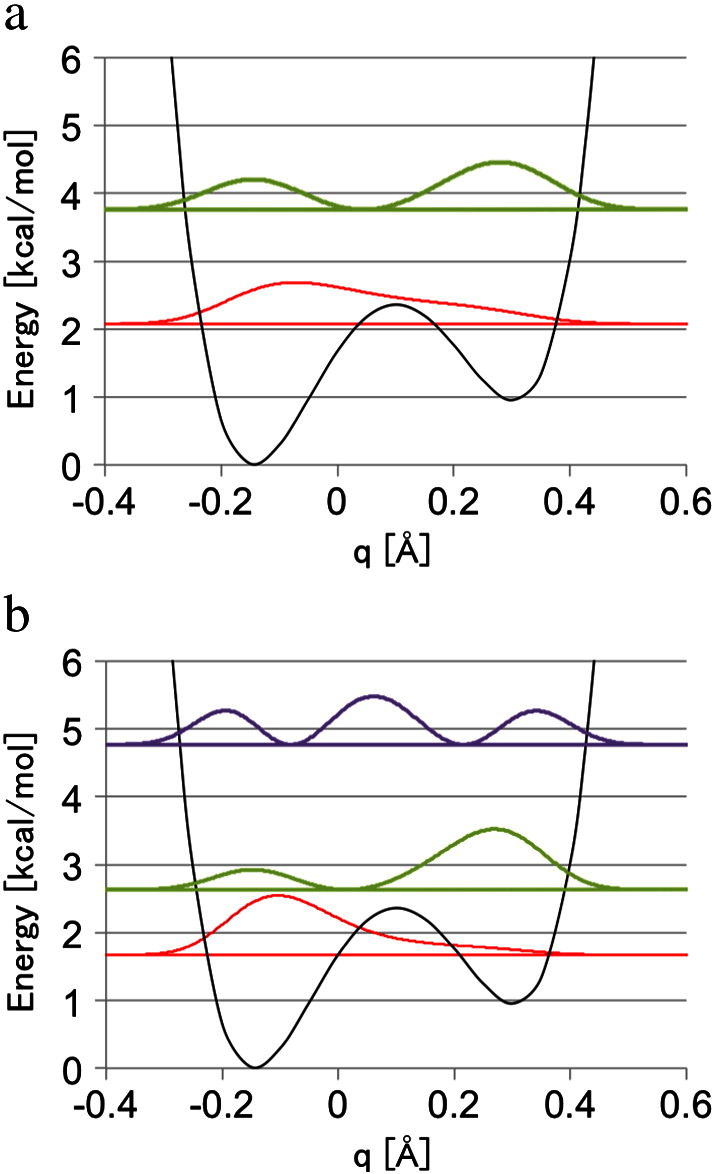
The potential energy curve (black) and the corresponding vibrational distribution of the ground (red) and the first (green) and second (purple) excited states of (a) a proton and (b) a deuteron in the hydrogen bond between Glu46 and pCA of PYP at the crystal structure. The origin of the coordinate *q* was set on the crystal bond length (1.21 Å).

Here, we would like to focus on the experimental crystal structure. Fig. 3 shows the potential energy curve and the corresponding vibrational distributions of the ground and the first excited states of a proton and a deuteron. For the deuteron, the vibrational distribution of the second excited state is also shown. The potential energy curve represents a double-well shape at the crystal structure with the O…O distance of 2.56 Å, as already mentioned. The barrier height is 2.36 kcal/mol at *q* = 0.10 Å, where the OH bond length is 1.31 Å. The corresponding energy levels and the vibrationally averaged OH bond lengths are shown in Table 3.

We can see that the zero-point vibrational energy for a proton is almost comparable with the barrier height, and the proton overcame the barrier to widely spread. On the other hand, there is an energy gap of about 1 kcal/mol between the barrier height and the zero-point energy of a deuteron, and the ground state deuteron tends to localize around the minimum. This isotopic difference of the vibration results in a difference of the average bond length of the ground state by 0.06 Å, which is three times larger than that of the equilibrium structure in Table 2.

Another point worth mentioning is that the energy gap between ground and the first excited states for the crystal structure is lower than those of the equilibrium structure. Particularly, the deuteron only has a gap of 0.96 kcal/mol, which allows vibrational excitation at room temperature. The thermally averaged OH bond length was extended from the ground state by 0.03 Å for the deuteron, while it did not change in the shown digits for the proton. In other words, the OH bond length for the proton elongated by 0.17 Å from the energy minimum due almost only to the nuclear quantum effect, while that for the deuteron elongated by 0.11 Å due to the nuclear quantum effect and by 0.03 Å due to the thermal effect. The resulting length for the deuteron agreed with the experimental value within the error of 0.01 Å.

Whereas reasonable agreement was obtained, it should be noted here that the present analysis against one-dimensional partial vibration neglected the fluctuations of the other atoms and the motion of the hydrogen nucleus coupled with them. It cannot be said that the present analysis is more accurate or realistic than the previous analyses, although the reasonability of the one-dimensional potential energy curve used in the present analysis has been confirmed [17]. Higher-dimensional vibrational analysis would be desirable to acquire more quantitative reliability.

Finally, let us consider the difference of the equilibrium and the crystal structures. As already mentioned, they significantly differ in the H-bonding O…O distance of Glu46 and pCA by about 0.1 Å, involving the drastic difference of the potential energy curve. The agreement between the present results for the equilibrium structure and our previous result by ONIOM (MC_QM:MM) implies that the underestimation of the OH bond length by ONIOM (MC_QM:MM) could be possibly associated with the underestimation of the H-bonding O…O distance, taking into account that the vibrational analysis on the crystal structure with large O…O distance reasonably reproduced the experimental OH bond length. The underestimation would be owing to the lack of the O…O stretching by the nuclear quantum or thermal effect. Nadal-Ferret et al. demonstrated that a significant fluctuation of the O…O distance can arise from the thermal effect [16]. The crystal structure can be regarded as a thermally averaged structure, which may provide better energy profile for the vibrational analysis in the present work. It should also be noted that the error in the equilibrium structure from the CAM-B3LYP functional for the evaluation of H-bonding distance is not negligible [24], although the functional was reasonably selected for the evaluation of the energy profile of proton migration by Nadal-Ferret et al. [16]

## 4 Conclusion

We analyzed the vibrational states of the hydrogen nucleus in a hydrogen bond of photoactive yellow protein (PYP). We found that the potential energy curve was a single-well shape for the equilibrium structure, while it was a double-well shape with low energy barrier for the crystal structure. The vibrationally averaged OH bond length reasonably agreed with that of the deuterated PYP. The isotopic difference of the temperature dependency was suggested by the Boltzmann themal averaging. It was implied that the extension of O…O distance by the quantum or thermal effect from the equilibrium structure would be indispensable for the formation of the law-barrier hydrogen bond with drastically extended OH bond length as seen in the neutron diffraction measurement.

## Figures and Tables

**Table 1:** The experimental and the computational OH bond lengths of Glu46 in the deuterated PYP from the previous works.

Experimental	(ref. [2])	*R*	1.21
Computational	(ref. [15])	*R*_eq_	1.02
		⟨R⟩	1.05
	(ref. [16])	*R*_eq_	1.05
		⟨R⟩	1.10
	(ref. [17])	*R*_eq_	1.08
		⟨R⟩	1.14

Units are in Å. For computational lengths, both the equilibrium (*R*_eq_) and the vibrationally averaged ⟨R⟩ values are shown.

**Table 2:** Vibrational energy levels (*ε* in kcal/mol) for the first three states and the averaged OH bond lengths (⟨R⟩ in Å) for a proton (H) and a deuteron (D) in hydrogen bond between Glu46 and pCA of PYP at the equilibrium structure.

	State	*ε*	⟨R⟩
H	2	10.14	1.21
	1	5.46	1.23
	0	2.02	1.17
		*R_T_*	1.17
D	2	6.52	1.22
	1	3.73	1.24
	0	1.47	1.15
		*R_T_*	1.15

The bond length at the energy minimum is 1.08 Å [17]. Thermally averaged bond lengths (*R_T_* in Å) at room temperature are also shown.

**Table 3:** Vibrational energy levels (*ε* in kcal/mol) for the first three states and the averaged OH bond lengths (⟨R⟩ in Å) for a proton (H) and a deuteron (D) in hydrogen bond between Glu46 and pCA of PYP at the crystal structure.

	State	*ε*	⟨R⟩
H	2	7.36	1.29
	1	3.76	1.34
	0	2.08	1.23
		*R_T_*	1.23
	2	4.77	1.28
D	1	2.63	1.39
	0	1.67	1.17
		*R_T_*	1.20

The bond length at the energy minimum is 1.06 Å. Thermally averaged bond lengths (*R_T_* in Å) at room temperature are also shown.
